# Comparison of T1wFLAIR and T1wTSE sequences in imaging the brain of small animals using high-field MRI

**DOI:** 10.1186/s13620-019-0145-5

**Published:** 2019-07-06

**Authors:** Chiara Bergamino, Séamus Hoey, Kenneth Waller, Cliona Skelly

**Affiliations:** 1 School of Veterinary Medicine Teaching Hospital, University College Dublin, Belfield, Dublin Ireland; 2 Department of Surgical Sciences, University of Wisconsin, Madison, WI 53706 USA

**Keywords:** T1wFLAIR, MRI, Brain, Small animals, Inversion recovery

## Abstract

**Background:**

T1w turbo spin echo (TSE) represents a fundamental sequence in magnetic resonance imaging (MRI) protocols investigating the brain. Recent human literature has reported T1w Fluid Attenuated Inversion Recovery’s (FLAIR’s), superiority to T1wTSE in relation to tissue contrast for grey-to-white matter (GM-WM) and lesion-to-WM, although conflicting results are reported concerning lesion detection.

To the author’s knowledge, T1wFLAIR has not been investigated in veterinary medicine. The aim of this prospective study was to determine quantitatively and qualitatively which sequence provides better overall better image quality both pre- and post-gadolinium.

**Results:**

Twenty-eight animals underwent MRI of the brain with T1wTSE and T1wFLAIR sequences performed with equivalent mean acquisition times. Quantitative assessment of the sequences was undertaken using contrast-to-noise (CNR) and signal-to-noise (SNR) ratios from predefined locations. T1wFLAIR provided a better CNR compared to T1wTSE, while T1wTSE provided better SNR due to the higher noise levels of T1wFLAIR images. Qualitative assessment of the sequences was performed using Visual Grading Analysis Scoring (VGAS) for a number of criteria by three observers on two separate occasions. T1wFLAIR performed better for cerebrospinal fluid (CSF) suppression, white-to-grey matter (WM-GM) and white matter-to-CSF (WM-to-CSF) definition in both pre- and post-contrast images whereas the T1wTSE sequence was less affected by noise levels. The individual parameter for overall image quality found no significant difference between the two sequences. However, the composite VGAS favored T1wFLAIR as the preferred sequence. Although case numbers were insufficient for statistical analysis, comparison of the sequences indicates that lesion definition and margination was better in T1wFLAIR pre-contrast images, however post-contrast lesion detection was almost equivalent between sequences with slightly better margination in the T1wTSE sequence.

**Conclusions:**

T1wFLAIR provides better CNR with better WM-GM and WM-CSF definition both pre- and post-contrast compared to T1wTSE albeit with a higher degree of noise; this was confirmed both quantitatively and qualitatively. Our results also suggest that T1wFLAIR is better for lesion detection and margination pre-contrast administration and sequences are relatively equivocal post-gadolinium administration although further research is required to determine the benefit that inversion recovery sequences make when investigating brain lesions in small animal MRI.

## Background

T1weighted turbo spin echo (T1wTSE) is considered a fundamental sequence in clinical small animal brain MRI imaging [1, 2]. It is characterized by short echo (TE) and repetition (TR) times and used mainly for anatomical reference.

T1weighted Fluid Attenuated Inversion Recovery (T1wFLAIR) is an inversion recovery (IR) turbo spin echo sequence that has been investigated in human medicine since 1985 [3]. Since inception, the sequence has demonstrated improved WM-GM and lesion-WM contrast, but the long acquisition time has limited its use in clinical settings. Multiple studies have highlighted improvements in the technical aspects of the sequence and investigated its clinical usefulness [4–10]. In 2000 Lee et al.*,* examined the inversion recovery sequence and determined that despite the longer acquisition time, T1wFLAIR improved the extent and conspicuity of lesions and was qualitatively superior for image contrast when compared to T1wTSE [6]. Human medical literature report overall agreement among different studies on the ability of T1wFLAIR in providing better WM-GM definition and better lesion-to-WM contrast in both high- and low-field MRI [3–7, 9, 11] and in both pre- and post-contrast studies [9, 12]. Some contradictory results have, however, been published regarding the ability of T1wFLAIR in detecting brain disease [8–10, 12]. The study conducted by Qian et al.*,* (2008), found a considerable number of lesions detected only on T1wTSE, in both pre and post-contrast sequences [8]. A more recent study by Jeong et al. (2014) evaluated the role of T1wFLAIR in oncologic patients and concluded that the inversion recovery sequence was better or comparable to T1wTSE both quantitatively and qualitatively in post-contrast imaging [10].

In the veterinary literature, one study compared the contrast-enhanced T2w FLAIR and T1wSE image sequences [13]. The study focused on the usefulness of T2wFLAIR compared to standard T1wSE in detecting brain lesions in post-contrast images in low-field magnets. It was reported that T2wFLAIR allowed the identification of a higher number of lesions compared to T1wSE. Falzone et al. (2008) concluded that the contribution that inversion recovery sequences made to small animal brain imaging required further investigation to assess its full potential [13].

In light of the conflicting reports regarding lesion detection in the human medical literature and in order to quantify the contribution that T1wFLAIR gives to brain imaging in terms of tissue contrast from a veterinary perspective, a prospective study was undertaken.

The study was designed to acquire images using parameters that would minimize the differences in the length of time for acquisition to increase the feasibility of using T1wFLAIR in a clinical setting. The post-acquisition image sequences were then analysed both quantitatively and qualitatively, pre and post contrast administration. The quantitative evaluation consisted of two objective assessments: contrast to noise ratio (CNR) and signal to noise ratio (SNR). Contrast refers to the signal difference between two tissues or a lesion and its background [14] and the CNR is defined as the ratio of signal difference (contrast) to the standard deviation of background noise (σ). SNR is defined as the ratio of the amplitude of the MR signal to the average amplitude of the background noise [14]. Both parameters are considered important for image quality.

Visual grade analysis (VGA) is a qualitative image assessment method that permits an image or parts of an image to be evaluated visually [15]. Using predefined criteria VGA allows the quantification of subjective opinions into a scoring system making them amenable to statistical analysis [16]. VGA has been validated in human radiology, as an analytical system for image quality [15, 17–19]. Currently, in veterinary medicine VGA has also been examined and it is now considered a valid option in radiology to quantitatively assess the image quality [20].

The primary aim of this prospective study is to quantitatively and qualitatively compare T1wTSE with T1wFLAIR images to determine which sequence provides better CSF suppression, GM-WM contrast, lower noise and overall better image quality both pre- and post-gadolinium. The hypothesis is that T1wFLAIR would provide better CSF suppression, GM-WM and WM-CSF contrast. The secondary aim of this study is to evaluate which image sequence would provide better image quality taking into account the influence of contrast to noise and signal to noise ratios. The study also compared the sequences for lesion detection and margination pre and post contrast enhancement.

## Methods

### Animals

This was a prospective, comparative study of 28 adult animals admitted to the Veterinary Teaching Hospital, University College Dublin and Veterinary Medical Teaching Hospital, University of Wisconsin-Madison undergoing MRI investigation of the brain between May 2017 and July 2018. T1wFLAIR and T1wTSE sequences formed part of the routine sequences for brain imaging at both institutions, allowing exemption from ethical review. Animals were included in the study when both T1wTSE and T1wFLAIR were performed within the same examination, in the same transverse plane and with the same slice thickness in both pre- and post-contrast statuses. A patient was excluded when the T1wFLAIR parameters were changed, when the two sequences were acquired only in pre- or post-contrast or when the two sequences had different slice thickness.

### Image acquisition

Images were acquired using two MRI scanners at each of the centres (1.5 T Philips, Achieva, Philips Medical System, The Netherlands, 1.5 T GE Genesis Signa, GE GE Healthcare Milwaukee, USA) using head or extremity coils, depending on the size of the animal. The MRI brain protocol included transverse T1wTSE and T1wFLAIR, acquired at the same slice thickness, adapted to the size of the patient (2.5–5 mm), both pre- and post-gadolinium (PG) administration. T1wTSE parameters were as follow: TE 9–12 ms (milliseconds); TR: 450–700 ms; NEX: 2–4. For T1wFLAIR parameters were: TE:16 ms; TR: 2000 ms; TI:600 ms; NEX: 2. The acquisition time for each sequence was recorded.

### Quantitative analysis

SNR for the white matter (WM), grey matter (GM) and cerebrospinal fluid (CSF) and CNR for the WM-GM and WM-CSF were measured in all 4 sequences (T1wTSE, T1wFLAIR, T1wTSE PG and T1wFLAIR PG). A standard circular region of interest (ROI) was drawn in a number of different areas of the central nervous system in all sequences by one board certified radiologist. The predefined ROIs chosen for WM were: the cerebellar hemisphere, the internal capsule and the thalamus. The GM ROIs were placed at the cingulate gyrus, temporal cortex and piriform lobe, alternating between the right and left side in structurally normal brains and choosing the unaffected site in cases with pathological changes. The mean of the three ROIs for WM and GM were used for statistical analysis. For the CSF ROI, the lateral ventricles were the preferred site, however, occasionally when they were too small to allocate the ROI, the third ventricle or the aqueduct was chosen instead.

### Qualitative analysis

Three board-certified radiologists independently evaluated the DICOM (Digital Imaging and Communication in Medicine) images in two separate sessions a minimum of 3 weeks apart. Cases were anonymized and identified with a progressive number, generated by a random number generator and each number was different between the two sessions. For each patient the pre-contrast sequences were identified as “A” or “B”, while the post-contrast as “C” or “D”. Observers were asked to compare the images in pairs (A with B, and C with D) evaluating each pair using post-processing alterations of the images (such as magnification or windowing) where appropriate. The qualitative criteria examined were: the degree of CSF suppression; WM-GM definition; WM-CSF definition; the presence of noise; presence of other artifacts affecting the region of interest (cerebrum, cerebellum, meninges and neurocranium) overall better image quality. Observers expressed their preferences choosing the letter (A or B, C or D) for each criteria of the paired sample. If neither sequence was preferred, then a value of 0 was given for equivalence. The data was then collated using a three-grade scale. The observer preferences were assigned a value, such that when T1wFLAIR was the preferred sequence, a score of + 1 was given, when T1wTSE was the preferred sequence a score of − 1 was given, and a 0 score was given if the two sequences were considered equal. Composite Visual Grade Analysis Score (VGAS) for each comparative set of sequence was calculated based on 4 of the 5 parameters measured (CSF suppression, WM-GM definition, WM-CSF definition and presence of noise). Overall image quality, as judged by the observers, was not included in calculation of the composite VGAS as it was an amalgam of the other criteria.

Observers also judged if a lesion was detectable on one or both sequences pre and post contrast. If present, the sequence which provided better lesion margination and detectability was indicated by the letter of the preferred sequence or a 0 in case of equivalence. The data was then collated using the same three-grade scale.

### Statistical analysis

Statistical analysis was carried out by a veterinary statistician.

### Quantitative analysis

Sample size calculation using G-Power was performed to find the minimum number of animals for the study. Given a two-tailed design with an alpha error = 0.0002 (due to the Bonferroni correction for multiple hypotheses) and effect size of 1, we can expect to identify significant differences with an 80% power with a sample size of 21. At sample size 28, the same power can be achieved for effect size ~ 0.82. Further, sample size of 28 is a reasonable size for the t-test to be valid should the data be non-normally distributed [21].

For the CNR, using STATA 15, a two-tailed t-test with 27 degrees of freedom was performed for each comparison for the null hypothesis that the mean of the ROIs for T1wFLAIR was equal to the mean of T1wTSE.

A sign test was carried out for the SNR, evaluating how the median value for the T1wFLAIR sequence rated against the median value of T1wTSE. A positive observation indicating T1wFLAIR performed better than T1wTSE, a negative observation that it performed worse, with a 0 ranking as equivalent.

### Qualitative analysis

A two-tailed t-test was performed on the mean VGAS for each of the 6 parameters in both pre- and post-contrast using the statistical package STATA 15.

To assess repeatability of measurements, agreement over sessions was calculated for each observer (intra-observer) using Gwets AC kappa, with linear weighting and probabilistic benchmarking. Gwets AC kappa was also used for the inter-observer agreement for each parameter using the mean value for each observer over the two sessions in order to calculate reproducibility. Landis and Koch (1977) suggest the following benchmark scale for interpreting the kappa-statistic: < 0.00 Poor; 0.00–0.20 Slight; 0.21–0.40 Fair; 0.41–0.60 Moderate; 0.61–0.80 Substantial; 0.81–1.00 Almost Perfect [22].

Due to the small number of lesions within the case cohort, descriptive statistics are presented. The cases were divided in pre- and post-contrast administration. Mean VGAS for lesion detection and lesion margination, respectively, were calculated for subjects where all three observers agreed that lesions were present. Mean VGAS was calculated using the grades for both sessions and all three observers. The results are presented as boxplots of the mean VGAS for lesion detection and margination pre and post contrast administration.

The Bonferroni correction for 25 hypotheses and a significance level of *p* <  0.05 was calculated and resulted in a significance level of 0.002.

## Results

### Animals

The study population was composed of 26 dogs (10 male neutered; 9 females spayed; 5 males; 2 females) and 2 cats (1 female spayed and 1 male neutered). The range of dog breeds included were: 3 Boxers, 2 German Shepherds, 2 Boston Terriers, 2 Terrier Crosses, 2 Golden Retrievers, 2 Bulldogs, 2 Maltese Terriers, and one Border Collie, Pitbull Terrier, Labrador, Australian Shepherd, Dachshund, Rottweiler, Cocker Spaniel, Miniature Poodle, Poodle Cross, Brussels Griffon and Cavalier King Charles Spaniel. Both of the cats included in the study were Domestic Short Hair. The mean weight in the population was 18.1 Kg (range: 3.5–40.5 Kg) and mean age was 7.2 years (range: 1–15.2 years). Brains were structurally normal in 17 cases, while abnormalities were found in 11 cases: 4 intra-axial masses; 3 extra-axial masses; 1 leptomeningitis; 1 optic neuritis; 1 otits interna and 1 leukariosis.

### Image acquisition

The mean acquisition time for T1wTSE was 4 mins (minutes) and 44 s (seconds) (Range: 2 mins 56 s – 6 mins 17 s) and the mean acquisition time for T1wFLAIR was 5 mins and 6 s (Range: 3 mins 38 s - 6 mins 03 s).

### Quantitative analysis

The results for the CNR for the sequences with and without Gadolinium are shown in Table 1. T1wFLAIR performed better than T1wTSE for CNR in all cases with the exception of WM-CSF contrast where no significant difference was found between the two sequences.

**Table 1:** Comparison of the Contrast to Noise Ratio (CNR) for T1wFLAIR and T1wTSE Sequences Pre and Post Contrast Administration

Measure	Gad	T1w FLAIR	T1wTSE	*P*-value^a^	SE	Bonferroni Adjusted confidence interval
WM-CSF	No	12.23	10.52	0.0008	0.45	0.18 3.23
WM-CSF	Yes	12.88	10.91	0.0048	0.64	−0.19 4.13
WM-GM	No	5.05	2.03	< 0.00001	0.30	2.01 4.03
WM-GM	Yes	4.55	1.75	< 0.00001	0.25	1.95 3.64

^a^ two-tailed t-test*: Gad* gadolinium

Results for SNR are summarized in Table 2. For all comparisons, except for the SNR of WM pre- and post-contrast, the null hypothesis was rejected proving that T1wTSE showed higher SNRs for GM and CSF in both pre- and post-contrast. However, there was not enough evidence to reject the null hypothesis for the WM in the pre- and post-contrast series based on the Bonferroni-adjusted significance level of 0.002, calculated from a significance level of 0.05 for 25 hypotheses.

**Table 2:** Comparison of the Median Signal to Noise Ratio (SNR) for T1wFLAIR verses T1wTSE Sequences Pre and Post Contrast Administration

		No. of Observations for T1wFLAIR verses T1wTSE			
Measure	Gad	Negative	Positive	Median of Differences Median	two-tailed *P*-value
SNR_CSF	No	25	3	- 4.91	0.00003
SNR_CSF	Yes	25	3	- 5.48	0.00003
SNR_GM	No	25	3	−5.57	0.00003
SNR_GM	Yes	24	4	−5.33	0.00018
SNR_WM	No	21	7	−2.96	0.01254
SNR_WM	Yes	22	6	−2.82	0.00372

*Gad* gadolinium

### Qualitative analysis

The two-tailed t-test results are shown in Table 3 for the pre-contrast and in Table 4 for the post-contrast series. Results indicate a significantly better performance of the T1wFLAIR in the suppression of CSF signal, improved WM-GM and WM-CSF definitions and composite VGAS in both pre- and post-contrast series. T1wTSE images showed a statistically significant reduction in the levels of noise compared to the T1wFLAIR images both pre and post-contrast. Regarding overall image quality, the difference between the two sequences was not statistically significant.

**Table 3:** Comparison of the VGAS mean values for pre-contrast images of T1wFLAIR and T1wTSE sequences of the brain using predefined criteria

Imaging Criteria	VGAS Mean Value	Standard Error	*P*-value *	Bonferroni Adjusted
CSF Suppression	0.95	0.04	< 0.00001	0.83 1.07
WM-GM	0.92	0.04	< 0.00001	0.79 1.04
WM-CSF	0.88	0.03	< 0.00001	0.78 0.98
Presence of Noise	−0.88	0.03	< 0.00001	−1.00 -0.77
Overall Better Image Quality	−0.089	0.10	0.39606	−0.43 0.26
Composite VGAS	0.47	0.02	< 0.00001	0.40 0.54

* *P*-value derived from two-tailed t-tests with 27 degrees of freedom

**Table 4:** Comparison of the VGAS mean values for post-contrast images of T1wFLAIR and T1wTSE sequences of the brain using predefined criteria

Imaging Criteria	Mean Value	Standard Error	*P*-value*	Bonferroni Adjusted
CSF Suppression	0.82	0.07	< 0.00001	0.59 1.05
WM-GM	0.78	0.06	< 0.00001	0.56 1.00
WM-CSF	0.74	0.05	< 0.00001	0.55 0.93
Presence of Noise	−0.76	0.07	< 0.00001	−1.00 -0.53
Overall Better Image Quality	−0.09	0.10	0.36290	−0.41 0.23
Composite VGAS	0.39	0.03	< 0.00001	0.27 0.52

* *P*-value derived from two-tailed t-tests with 27 degrees of freedom

The mean of the observers (Table 5) for intra-observer reliability was ‘almost perfect’ for pre-contrast CSF signal suppression, WM-GM and WM-CSF definitions and presence of noise; ‘substantial’ for the post-contrast CSF suppression, WM-GM and WM-CSF definitions and presence of noise; ‘moderate’ for the post-contrast overall image quality and ‘fair’ post-contrast overall better image quality.

**Table 5:** Results for mean intra-observers agreement considering all observers assessing T1wFLAIR and T1wTSE sequences of the brain of dogs and cats

	Pre-contrast		Post-contrast	
Imaging Criteria	AC Gwet kappa	Probabilistic Benchmark	AC Gwet kappa	Probabilistic Benchmark
CSF Suppression	0.97	0.8–1	0.86	0.6–0.8
WM-GM	0.97	0.8–1	0.84	0.6–0.8
WM-CSF	0.89	0.8–1	0.79	0.6–0.8
Presence of Noise	0.90	0.8–1	0.76	0.6–0.8
Overall Better Image Quality	0.52	0.2–0.4	0.59	0.4–0.6

The overall inter-observer reliability (Table 6) for CSF signal suppression, WM-GM definition and presence of noise in the pre-contrast images was ‘almost perfect’ in pre contrast series, and ‘substantial’ in the post-contrast series. The inter-rater agreement was ‘moderate’ for both pre- and post-contrast WM-CSF definition with the overall better image quality parameter having ‘slight’ pre-contrast and ‘poor’ post-contrast reliability.

**Table 6:** Results for mean inter-observers agreement considering all observers assessing T1wFLAIR and T1wTSE sequences of the brain of dogs and cats

	Pre-contrast		Post-contrast	
Variable	AC Gwet kappa	Probabilistic Benchmark	AC Gwet kappa	Probabilistic Benchmark
CSF Suppression	0.96	0.8–1	0.86	0.6–0.8
WM-GM	0.94	0.8–1	0.81	0.6–0.8
WM-CSF	0.86	0.6–0.8	0.69	0.4–0.6
Presence of Noise	0.89	0.8–1	0.76	0.6–0.8
Overall Better Image Quality	0.17	0–0.2	0.1	0

The most frequently reported artifact for both sequences was magnetic susceptibility affecting over 80% of the pre (125/156) and post T1wFLAIR (127/156) sequence images and almost 20% of the post contrast T1wTSE (31/156). Chemical shift was noted in 13% of T1wFLAIR (20/156) and 10% of T1wTSE (16/156) images post contrast. Other artifacts recorded were: flow artifact, truncation artifact, wrap around artifact and volume averaging, and these affected less than 4% of the cases per image sequence with no predilection for either sequence.

**Fig. 1:**
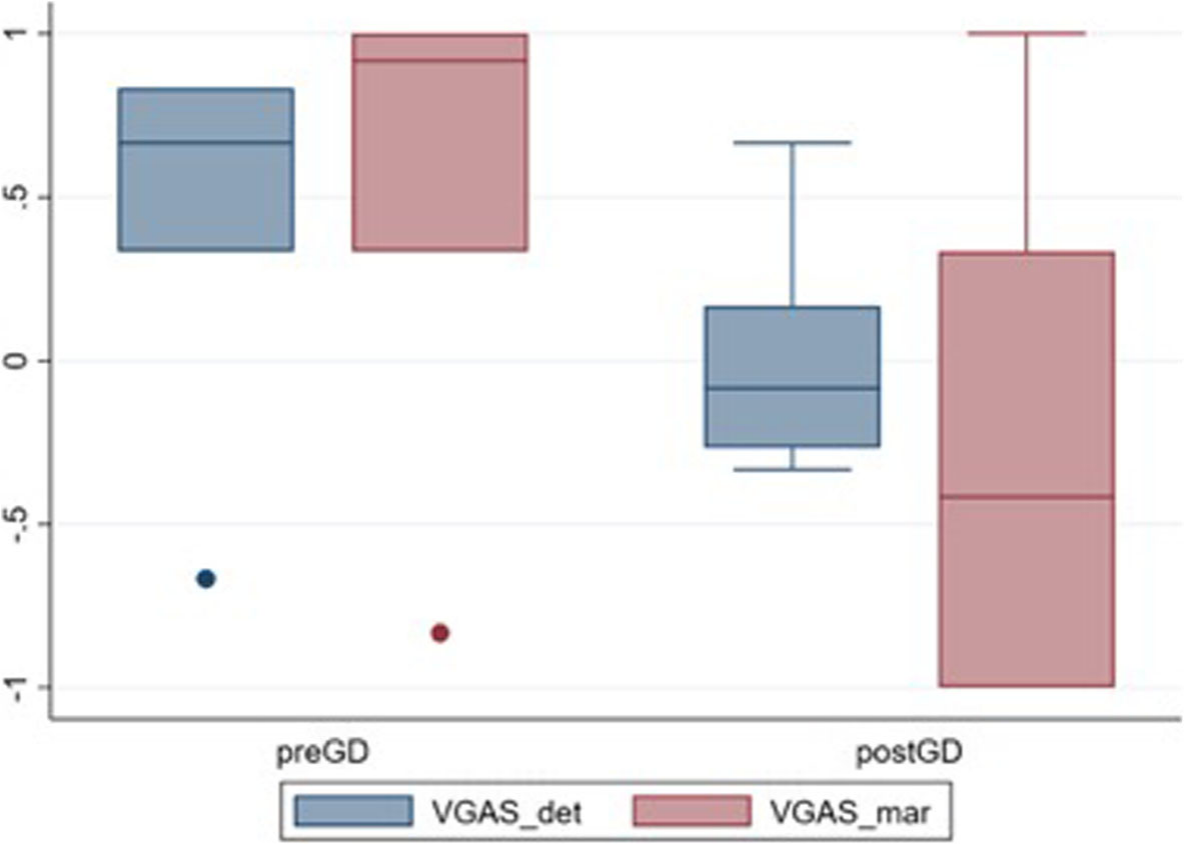
Boxplots of Composite VGAS for the lesion criteria including both sessions, for all three observers’ agreement. In pre-contrast images the three observers agreed on the presence of lesions in 6 cases out of 11; while in post-contrast images a full agreement was reached in 8 cases. Blue is detectability (det) and red is margination (mar); preGD is pre-contrast status; postGD is post-contrast. The three-grade scale is represented as + 1 (T1wFLAIR better); 0 (equivalent); −1 (T1wTSE better)

Regarding the lesion analysis, all three observers agreed that lesions were present in both sessions in 6 pre-contrast and in 8 post-contrast sets of images out of 11 abnormal cases. Considering only these cases in which all three observers agreed lesions were present, the distribution of results is represented in Fig. 1. Pre-contrast mean VGAS for detectability was 0.44, median 0.67; mean VGAS for margination was 0.56 median 0.92. Post-contrast mean VGAS for detectability was 0.00 median, − 0.08; mean VGAS for margination was − 0.27, median − 0.42 As shown in the box plots (Fig. 1), T1wFLAIR images provided improved lesion detectability and margination compared to T1wTSE in pre-contrast studies in a higher number of cases. However, on the post-contrast images the lesion detectability was almost equivalent, while margination was judged to be improved in the T1wTSE.

## Discussion

MRI sequence selection is based on providing tissue contrast to enhance the image quality of the area under investigation and enable more accurate interpretation diagnostically. In terms of the brain, the contrast between different brain tissues and the CSF is of primary importance. One of the strengths of the T1wFLAIR sequence reported in human literature is its superior image contrast [3–6, 9, 12]. In veterinary imaging there are a number of other key factors that need to be taken into account when assessing the image quality. Firstly, the human brain has a greater mass – so the lower SNR ratio in T1wFLAIR images would be expected to have a greater negative impact on the image quality of animal brains. Secondly, the shape of the canine brain is relatively longer in a rostro-caudal direction with relatively narrow olfactory bulbs further reducing the signal generated. In addition, the air-filled frontal sinuses and tympanic bullae that lie adjacent to the brain tissue provide conditions favouring high levels of magnetic susceptibility artifact that could adversely affect images in T1wFLAIR sequences. Finally, many of the human studies used extended T1wFLAIR acquisition times that are not clinically appropriate, thus preselecting parameters that minimized differences in acquisition times was important when comparing the sequences.

This study confirmed previously described superior CNR for T1wFLAIR images compared to T1wTSE demonstrating significantly higher GM-WM and WM-CSF CNRs for the T1wFLAIR [3–7, 9, 11, 12], in both pre- and post-contrast imaging of the brain of dogs and cats quantitatively (Table 1).

**Fig. 2:**
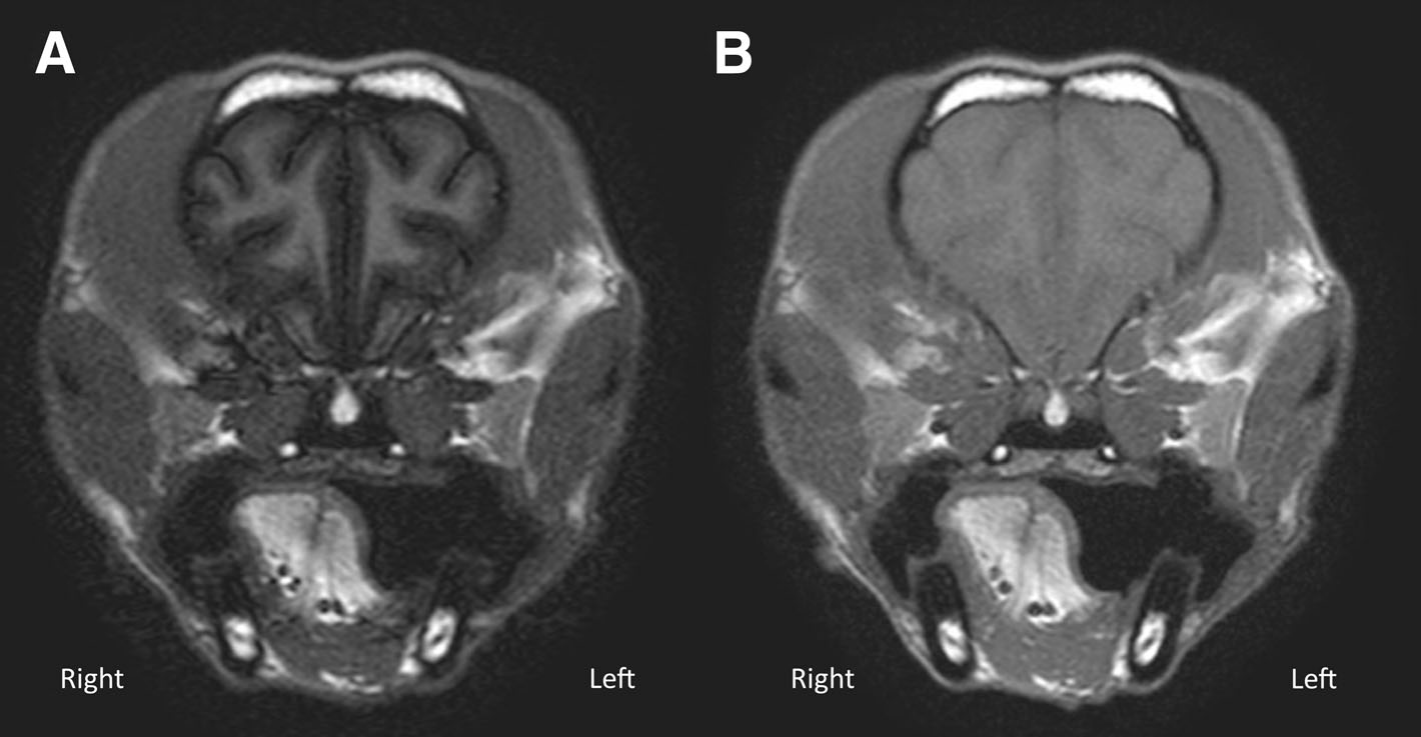
**a** Transverse T1wFLAIR (TE/TR/TI: 16 ms/2000 ms/600 ms; NEX: 2. ms; slice thickness 3 mm; Window Level: 797; Window Width: 1887); **b**) Transverse T1wTSE (TE/TR: 11 ms/470 ms; NEX: 3. ms; slice thickness 3 mm; Window Level: 797; Window Width: 1887) at the level of the frontal lobe of a small dog. T1wFLAIR (**a**) shows a better WM-GM contrast and a greater noise compared to T1wTSE (**b**). Dorsal aspect of the animal to the top of the image

The superior tissue contrast displayed in the T1wFLAIR sequence is due to the use of the characteristic 180° pulse (inversion pulse) [3, 9]. When applying the inversion pulse, the longitudinal magnetization becomes negative, increasing the contrast twofold during T1w relaxation [9]. Our results confirmed that despite any relative reduction in brain mass in animals compared to humans and the resultant reduction in SNR, that qualitatively T1wFLAIR retained its superior tissue contrast when using VGAS (Tables 3 and 4). The VGAS showed a statistically significant difference with better T1wFLAIR performance in both WM-GM and WM-CSF definition (Tables 3, 4 and 5) by all observers. The marked contrast definition is evident in Fig. 2, which shows the excellent differentiation of GM and WM of T1wFLAIR images, is considered an important parameter in MRI image assessment [23]. The high levels of inter- and intra- observer agreement, scoring the T1wFLAIR sequence as better in comparison to T1wTSE validates the superior tissue contrast of T1wFLAIR. Although gadolinium improved the overall contrast for the T1wTSE sequence, T1wFLAIR still remained superior to a ‘substantial’ and ‘moderate’ level in the inter-observer agreement analysis demonstrating that T1wFLAIR to provides better WM-GM contrast both pre- and post-gadolinium administration.

Considering the quantitative analysis, the only parameter that didn’t reach a statistically significance level was the CNR for the WM-CSF definition. However, as VGAS judged T1wFLAIR superior compared to T1wTSE for this parameter, it is likely that statistical significance was not reached due to insufficient case numbers.

While tissue contrast is considered a strength of the T1wFLAIR sequence, the reduced SNR is a recognized weakness. Comparing the two sequences, the SNR values were unsurprisingly significantly lower in T1wFLAIR than T1wTSE, with the exception of the white matter in post-contrast. For this variable the number of cases was likely insufficient to reach a significance. Overall the SNR results are in accordance with previous human studies [5, 11] despite the decrease in mass and different shape of the animal brain and demonstrate a similar strong reduction in signal relative to noise negatively impacting image quality. Although the majority (25 animals) in our case population demonstrated the lower SNR in the T1wFLAIR image sequence compared to that of T1wTSE, there were three animals (a cat, a Maltese terrier and a Boxer dog) with quite marked and unexpectedly high SNR values in the pre- and post-contrast T1wFLAIR sequence although their T1wTSE SNR values were as expected. There was no obvious reason to explain this finding as although the Phillips MRI machine performed all three studies, they used different coils and had different slice thickness (2.5 mm, 3 mm and 4 mm). In addition, a second Boxer dog and Maltese terrier replicated the MRI scan using the same machine and parameters and recorded the expected lower T1wFLAIR SNR levels. Therefore, as these three outliers would alter the mean SNR value for the T1wFLAIR sequence possibly leading to an erroneous interpretation of the result, it was considered more prudent to analyze the SNR data using Wilcoxon signed rank test, that does not assume that the data follows a normal distribution [24]. The results (Table 2) showed better SNR for the T1wTSE compared to T1wFLAIR is in keeping with the literature that IR sequences, such as T1wFLAIR, have lower signal-to-noise efficiency compared to TSE [25].

NEX (number of excitations) represents the number of times the signal originating from a certain slice is recorded [26]. The decision to use of a NEX of 2 for the T1wFLAIR as opposed to 2–4 for the T1wTSE was in order to maintain a similar acquisition time for the sequences. As a result, by lowering the NEX, the amount of signal recorded is decreased, however the scan time is also decreased [26]. In this study, a lower NEX in the T1wFLAIR sequence negatively impacted the signal recorded for the image sequence, meaning a higher level of noise in the displayed image, but did result in similar acquisition times when compared to T1wTSE, with a mean difference of only 22 s, representing a more clinically applicable comparison.

In light of the poorer SNR and lower NEX, the noise was as expected more conspicuous in the T1wFLAIR when compared to the TSE (Tables 3, 4) with inter-observers agreement reporting between ‘substantial’ to ‘almost perfect’ higher noise levels in the IR sequence in both sessions (Tables 5 and 6). High levels of noise give undesired signal that does not contribute to the image formation generated by fluctuations of the signal intensity [14, 23, 27]. There are two types of noise present in MR images. ‘Gaussian noise’, which was the predominant type found in this study, is responsible for quantum mottle and is displayed as a grainy pattern in the background. The second type is the ‘structured noise’, which is responsible for lines and streaks formation [14]. Both types of noise can adversely affect image interpretation by masking low-contrast lesions. So even though the noise level was greater in the T1wFLAIR images the inherent better contrast of the sequence minimized the negative impact on the images and this theory was supported by the VGAS preference for the T1wFLAIR image sequence.

**Fig. 3:**
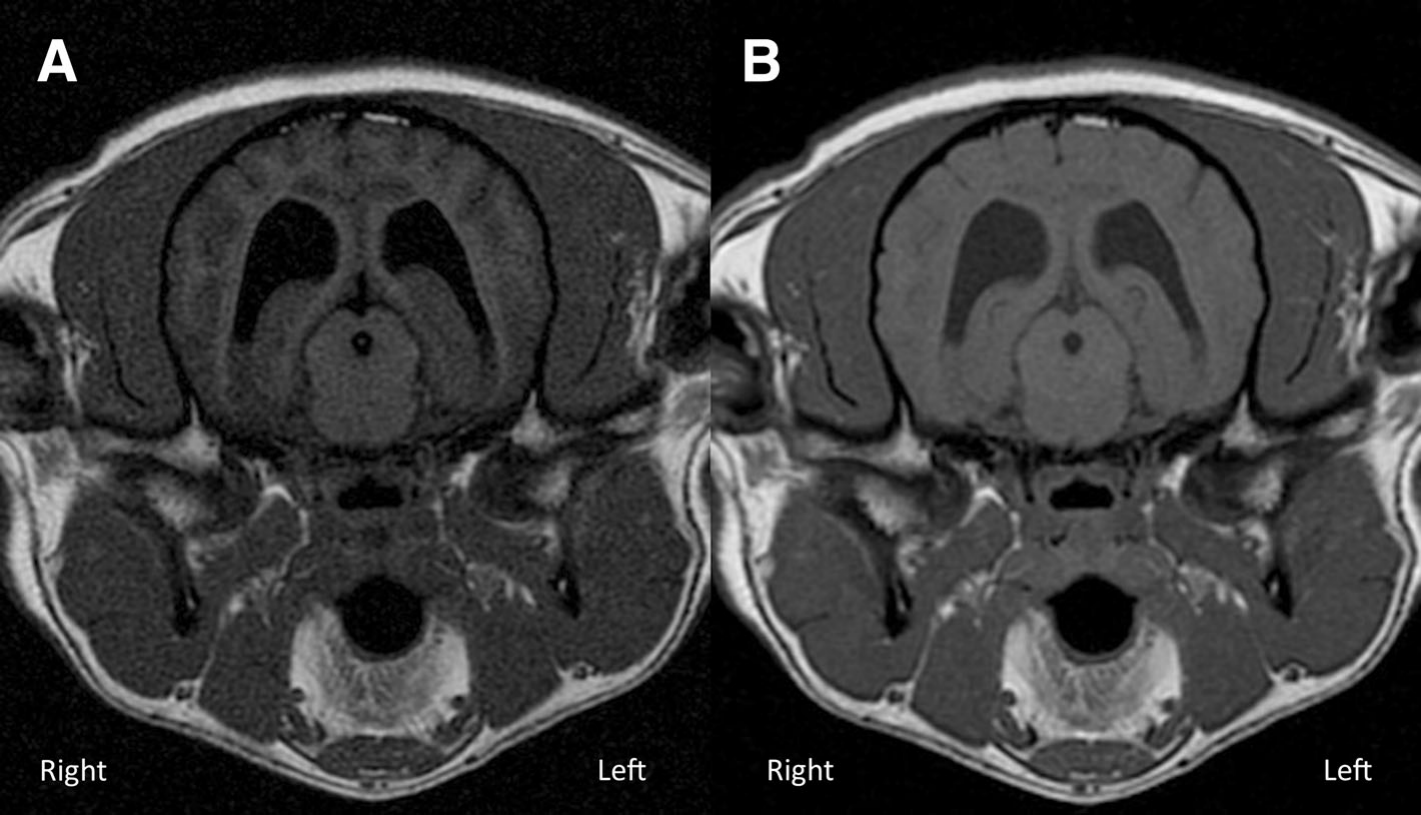
**a** Transverse T1wFLAIR (TE/TR/TI: 16 ms/2000 ms/600 ms; NEX: 2. ms; slice thickness 3 mm; Window Level: 1359; Window Width: 2613); **b**) Transverse T1wTSE (TE/TR: 11 ms/470 ms; NEX: 3. ms; slice thickness 3 mm; Window Level: 1359; Window Width: 2613) at the level of the thalamus in a medium size dog. T1wFLAIR (**a**) shows a better CSF signal suppression compared to T1wTSE (**b**). In (**a**) there is also better WM-CSF and GM-WM contrast in a compared to **b**. Note the higher level of noise affecting the T1wFLAIR (**a**). Dorsal aspect of the animal to the top of the image

Analyzing CSF signal suppression, both quantitative and qualitative analysis showed that the suppression in the T1wFLAIR sequence was significantly better than in the T1wTSE one (Fig. 3). This result represented a major advantage of the sequence, as the FLAIR allows the signal originating from the CSF to be nullified, contributing to a greater image contrast. The repeated inter-observer score varying between ‘almost perfect’ and ‘substantial’ in pre-contrast and post-contrast images indicate the repeatability and reliability of this result. CSF suppression can be advantageous in case of lesions in the subarachnoid space or intra-ventricular tumors or more in general in case of lesions located close to the CSF [28]. The nullification of the signal originating from the fluid adjacent/surrounding the lesion can improve its visualization [28].

Excluding noise there were a number of artifacts that affected image quality with T1wTSE images less affected in comparison to those of T1w FLAIR. Magnetic susceptibility was the most frequently observed artifact affecting over 80% of the T1wFLAIR pre and post contrast sequences compared to less that 20% of the T1wTSE ones. This artifact was reported by all observers affecting the rostral or caudal aspects of the skull, respectively at the level of the frontal sinuses, the olfactory bulbs and at the caudal fossa. In the human literature [5, 6] magnetic susceptibility has been reported, mainly but not exclusively in patients with metallic implants. The impact of this artifact can be reduced by the use of a longer echo train length; however, this would result in an increase in ghosting and blurring artifacts that also compromise image quality [5]. Other artifacts (chemical shift, flow artifact, Truncation artifact, wrap around artifact and volume averaging) were present but had no significant difference between sequences.

The qualitative results of the study were examined in two ways as a composite VGAS and a VGA of ‘overall image quality’. The composite VGAS was an amalgam of all the variables excluding ‘overall image quality’, considering each parameter equal in importance e.g. GM-WM contrast, CSF suppression etc., and their contribution to image quality. ‘Overall image quality’ was based on personal judgement of the image and each observer was likely to attribute a different significance to each parameter, leading to a less objective evaluation compared to the composite VGAS. While the composite VGAS demonstrated T1wFLAIR to provide significantly better image quality (Tables 3 and 4), the t-test results for the VGA score for the single parameter “overall image quality”, by the observers showed no statistically significant difference between the sequences in either pre-contrast or post-contrast studies. This discrepancy in the results may be that despite the superior contrast of the brain tissue in the T1wFLAIR sequence as evidenced by the composite VGAS, the increased levels of noise and the greater incidence of the magnetic susceptibility artifact rendered the images less visually appealing. Thus, observers judged the image quality to be equivocal between T1wFLAIR and T1wTSE and the marginal difference in the “overall image quality” between the sequences is highlighted by the ‘poor’ inter-observer agreement for both pre and post-contrast images. This was reinforced by the ‘moderate’ intraobserver agreement, indicating that the observers themselves regularly changed their preferred sequence highlighting the negligible difference in ‘overall image quality’.

The secondary aim of the study was to investigate the effectiveness of the IR sequence in detecting intracranial lesions. Although insufficient case numbers with lesions were present in the study to permit statistical analysis, there were sufficient numbers to highlight trends in the data. The results suggest that administration of a contrast media improves lesion detection in both sequences with 6 cases being visualized in both pre-contrast studies compared to 8 in the post contrast ones. According to our results, pre-contrast T1wFLAIR imaging provided both better lesion definition and margination when compared to standard T1wTSE; however, after contrast administration the detection was almost equivalent between sequences and slightly improved margination in the T1wTSE was reported (Fig. 1). This is partially in conflict with Qian et al (2008) which indicated the inversion recovery as the sequence providing higher lesions’ contrast enhancement [8]. This discrepancy may be due to the different pathologies included in the two studies, as Qian et al only investigated neoplastic lesions [8]. The current study results partially agree with Lee et al*’s* study (2000); that showed T1wFLAIR equivalent or superior for lesion detectability compared to T1wTSE in pre contrast images but differs from this study as they found post-contrast T1wFLAIR’s superiority was maintained [6]. Further research in which a greater number of intra-cranial lesions are present should the considered to allow statistical analysis, although the results may be inconclusive as it remains controversial even in the human medical literature [8–10, 12].

## Conclusion

T1wFLAIR demonstrated superior contrast of brain tissue when compared to T1wTSE both by quantitative and qualitative assessment in MRI veterinary imaging of the brain of dogs and cats. The IR sequence definitively showed better CSF suppression, WM-GM and WM-CSF definition pre- and post-contrast administration when compared to T1wTSE. The results also suggest that T1wFLAIR may be helpful in detecting intra-cranial lesions especially in pre-contrast images as they showed better lesion detectability and margination although further studies are required with increased case numbers to statistically confirm this hypothesis.

Hence, T1wFLAIR is a useful adjunctive sequence when investigating the brain of dogs and cats and may be more effective in pre contrast lesion detection.

## Data Availability

All data generated for this study are included in the presented article.
